# Cytology and molecular study for GSTP1 effect on asthma Iraqi patients

**DOI:** 10.1186/s12948-019-0108-0

**Published:** 2019-03-05

**Authors:** Israa Hussein Hamzah, Farha A. Ali Shafi, Sahar A. H. Al Sharqi, Suaad Almas Brakhas

**Affiliations:** 1 Biology Department, College of Science, Mustansiriyah University, Baghdad, Iraq; 2 Allergy Specialized Center, Baghdad/Resafa, Iraq

**Keywords:** Asthma, GSTP1, Polymorphism, BsmA1, RFLP, Iraq

## Abstract

**Background:**

GST belongs to a super family of phase II detoxification enzyme and it plays an important role in preventing the damage that may occur due to reactive water-soluble compounds generated by the association of reactive intermediates with glutathione.

**Method:**

In the present study, we analyzed the frequencies of GSTP1 polymorphism among the Iraqi population using PCR–RFLP technique. Fifty samples from bronchial asthma patients and fifty samples from control cases were subjected to conventional PCR and Restriction Fragment Length Polymorphism (RFLP) to detect GSTP1 genotype and measured different parameters together such as IgE, eosinophilic count, WBC, and so forth. Some of the cases were made to undergo sequence analysis and enrolled in NCBI GenBank with accession number (MG657249–MG657258). The GSTP1 polymorphism was determined using PCR and the resultant 176-bp fragment was subjected to RFLP and digested with BsamA1 to recognize the A–G transition at nucleotide.

**Results:**

Homozygotes for Ile105 encoding allele resulted in 176-bp fragment found in 62% andVal105 encoding allele had two fragments of 91 and 85 bp in PCR was found in 4% of asthmatic patients. On the other hand, heterozygotes resulted in three fragments of 176, 91 and 85 bp seen in 34% of patients.

**Conclusion:**

To the best of the researcher’s knowledge, this is the first-of-its-kind report with regards to the role played by GSTP1 polymorphism in bronchial asthma among the Iraqi patients. Though the study outcomes do not support the large role played by GSTP1 gene polymorphism in the evolution of bronchial asthma disorder, future researchers are suggested to investigate more features for many promising results.

## Introduction

Asthma occurs as a result of continuous irritation of the accomplishing areas in lungs (in particular, the bronchi and the bronchioles) which subsequently results in expanded contractibility of the encompassing smooth muscle groups [1–5]. Asthma has an effect on people irrespective of their age though it most affects during the early stage of life. It has a high rate of occurrence across the world in the past 25 years [2]. The pathogenesis and etiology of asthma are very complex and not completely understood. It is a far prediction that asthma is caused by a combination of genetic and environmental factors which includes exposure to air pollutants and allergens. Asthma may occur due to other capacity triggers such as medicinal drugs like aspirin and beta blockers [3–6].

Glutathione S-transferase P (GST), encoded by the GSTP1 gene, is a human enzyme that predominantly shields the human body against antioxidants and play an important role in the regulation of inflammatory responses. GST usually conjugate glutathione with electrophilic materials that are capable of generating free radicals as a result of detoxification [7–9]. The GSTP1 gene is positioned in the chromosome 11q13 whereas the polymorphisms of GSTP1 gene act as danger elements for asthma disease especially four alleles such as GSTP1A (Ile105–Ala114) GSTP1B (Val105–Ala114), GSTP1C (Val105–Val114) and GSTP1D (Ile105–Val114) [9–11].

There is no or ignorable data on gene polymorphisms of GSTP1 and their role among asthma patients from Iraqi providence. Therefore we intend to design this work with speculation that GSTP1 polymorphism has a role to play in asthma related phenotype. This study also discovers any association of blood indices with asthma patients.

## Materials and methods

This study was designed and approved by the Institutional Ethical Committee. According to university hospital protocol, bronchial asthma was diagnosed primarily based on medical signs, symptoms, and response to remedy as per the Global Initiative on Asthma classification scheme [12, 13]. The study was done among 100 Iraqi individual consisting 50 clinically-diagnosed asthma patients and 50 healthy controls from the Allergy Specialized Center, Baghdad/Resafa. Their age range was 16–65 years. Subsequent elaborate data was obtainedwhich included age, gender, private history of allergies, seasonal coryza, eczema, allergic conjunctivitis and total serum (IgE level > 100 IU/ml) measured by Enzyme-Linked—Immunosorbent Serological Assay (ELISA) whereas the White Blood Cells (WBC), HB, ESRand eosinophil count were calculated by victimization machine—driven count system.

### Sample collection

Five ml of blood sample was collected from all the study participants including control by vein puncture using disposable syringes. The blood sample was aliquoted into two tubes of which the first was to obtain a clot which was then separated by centrifugation at 3000 rpm for 10 min to separate serum. This was used for IgE level measurement assay through ELISA. The second aliquot of 2 ml was kept in EDTA tube, and stored in the freezer (− 20 °C) in order to extract DNA. The total IgE kit was used to estimate the IgE class antibodies level in the serum of patients and control (Total IgE ELISA Kit Quantitative Assay for Total IgE Antibodies, Omega diagnostic).

### Molecular analysis

For PCR and RFLP analysis, the DNA was extracted from whole blood using Wizard Genomic DNA purification kit (Promega) according to the manufacturer’s instructions. The GSTP1 polymorphism was by PCR-based Restriction Fragment Length Polymorphism (PCR–RFLP) according to a published protocol [14]. The sequence of primer that targeted GSTP1 is as follows F: ACCCCAGGGCTCTATGGGAA; R: TGAGGGCACAAGAAGCCCCT. The extracted DNA was used as a DNA template in a 25 µl solution containing 12.5 µl of Tag^®^Green Master Mix (2×) (Promega, USA), 1.5 µl each from forward and reverse primers, 5 µl of DNA template and 4.5 µl of nuclease-free water. The PCR condition was as follows: an initial denaturation step at 95 °C for 5 min followed by 30 repetitive cycles of denaturation at 94 °C for the 30 s, annealing at 60 °C for 30 s and extension at 72 °C for 30 s. A final extension at 72 °C for 10 min was also included. After that, the PCR products were purified with the commercially available kit (PCR Clean-up Kit, USA) to remove the excess dNTPs, primers, ethidium bromide, and enzymes.

The purified products of PCR were digested in 20 µl quantity for 2 h at 55 °C with 2.5 BsmA1 (BioLab). The digested products were then checked in 3% agarose gel stained with ethidium bromide. The presence of 176 bp fragment indicated the wild-kind genotype (Ile/Ile) while the presence of 85 and 91 bp fragments indicated the homozygous polymorphic genotype (Val/Val). The heterozygote turned into records in the presence of all the three fragments. Negative and positive controls were protected in all reactions to ensure that the samples are not contaminated during the procedures.

The sequences of all amplified DNA of patients blood samples retrieved from the DNA sequencer were then submitted to three international bioinformatics websites such as NCBI gene bank (National Center for Biotechnology Information), DNA data bank of Japan (DDBJ) and European bioinformatics institute (EMBL) with same title of this article along with a unique ID number (Accession number) (MG657249–MG657258). The statistical analysis was performed using the SPSS software program with P-values < 0.05 as the significance. The descriptive records were expressed as the mean ± standard deviation (SD)[15].

## Results

The individuals were recognized whether they are asthmatic or non-asthmatic on the basis of allergic or respiration signs, pores, skin allergic reaction assessments, IgE tiers, lung feature indices and other different signs such as wheezing, cough, dyspnea, and/or chest tightness. Among the 100 subjects who participated in this study, 21 (42%) were male asthmatics and 29 (58%) were female asthmatics, while the Male-To-Female ratio inside the control group got changed into 23 (46%) and 27 (54%) respectively (Table 1).

**Table 1:** The classify of gender for the studied cases and controls

	Gender distribution				
	Frequency (n)	Percent	Valid percent	Cumulative percent	Significance (P)
Cases					
Male	21	42	42	42	0.0572
Female	29	58	58	100	
Total	50	100	100		
Control					
Male	23	46	46	46	0.0572
Female	27	54	54	100	
Total	50	100	100		

The mean age of the considered cases was 44.10 ± 11.744 while the mean age of females was 43.58 ± 12.347 and the overall age range was 16–65 years (Table 2). The private characteristics of the studied instances and controls are summarized in Table 3. Eosinophilic count, WBC, HB, and ESR confirmed that there is no statistical significance of the distinction between the cases and the controls while IgE and the history of individual asthmatic patients showed a significant difference.

**Table 2:** The personal characteristics according age and gender

Personal characteristics	Cases (n = 50)	Control (n = 50)	Significance (P)
Sex (male)	42%	46%	0.0258
Sex (female)	58%	54%	
Age (years) (minimum–maximum)	17–62	16–65	0.0572
Range	45	49	
Mean ± SD	44.10 ± 11.744	43.58 ± 12.347

**Table 3:** The laboratory and pulmonary function tests results of the studied samples with bronchial asthma according to GSTP1 genotyping

Laboratory/pulmonary function results	Patients sample		Control		Significant (P)
	Mean	SD	Mean	SD	
WBC 10^9^/l	9.218	± 3.10350	8.76	± 2.49816	0.05
HB	12.3	± 1.52610	12.5	± 1.66831	0.05
ESR	24.26	±15.10347	20.16	± 8.73034	0.05
Eosinophil	5.06	± 2.14200	4.48	± 1.432	0.05
IgE	311.39	± 135.98607	68.23	± 30.16270	0.05
Family history					
Yes	44	NA	2	NA	
No	6	NA	48	NA	

**Fig. 1:**
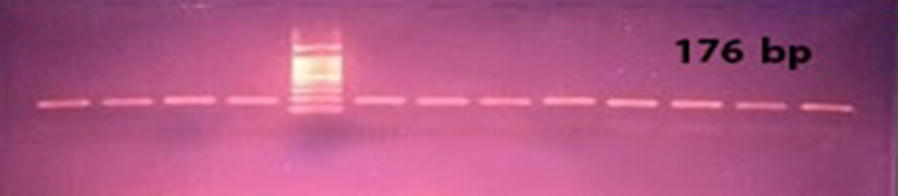
The product of PCR

**Fig. 2:**
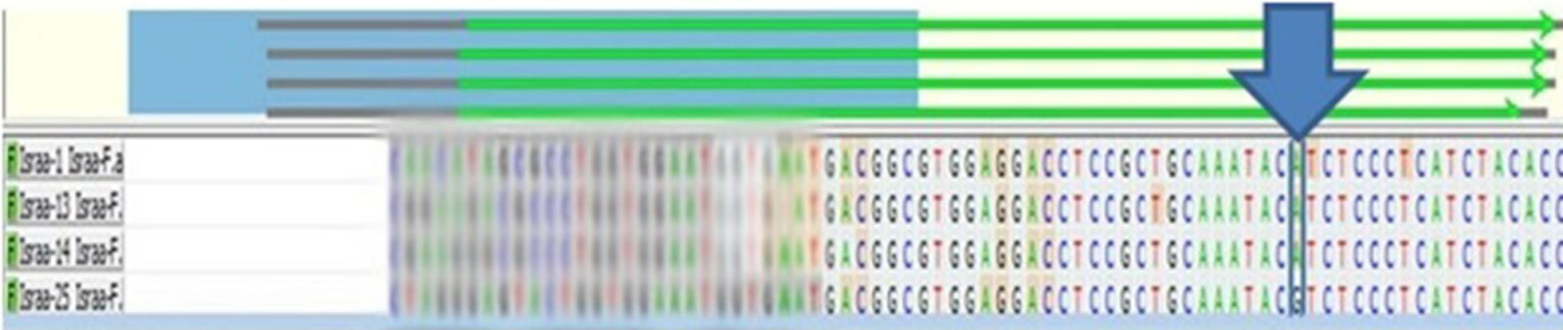
The analysis of the A–G transition at nucleotide

**Fig. 3:**
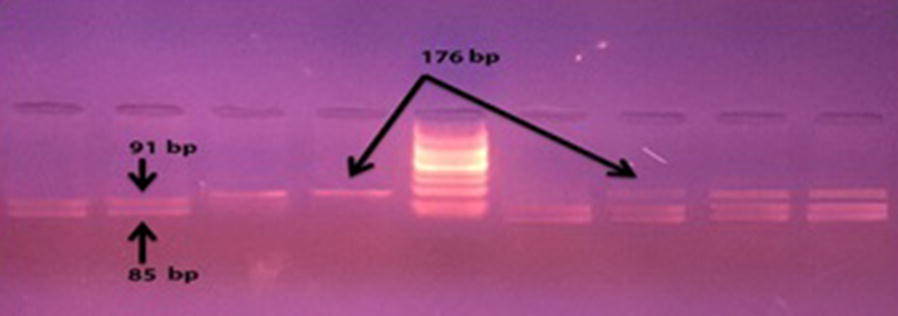
The results of REFLP

**Fig. 4:**
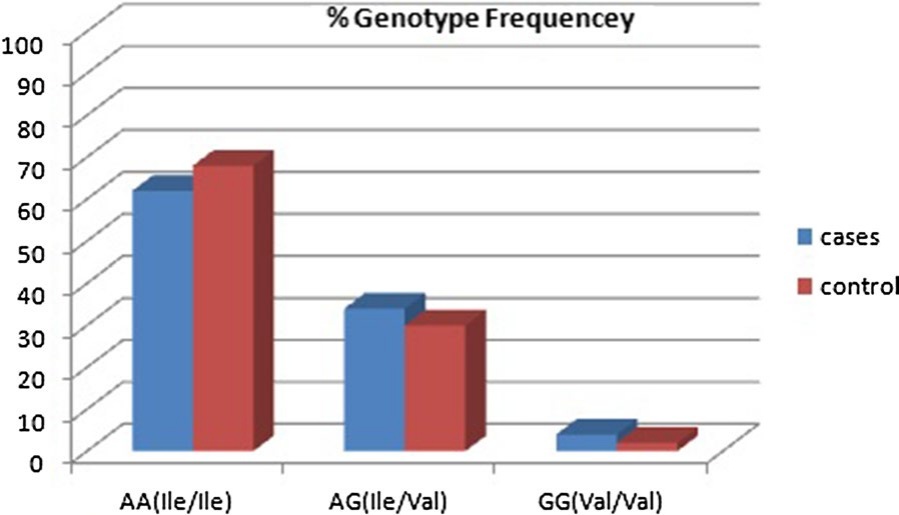
The genotype frequencies of GSTP1 alleles

**Fig. 5:**
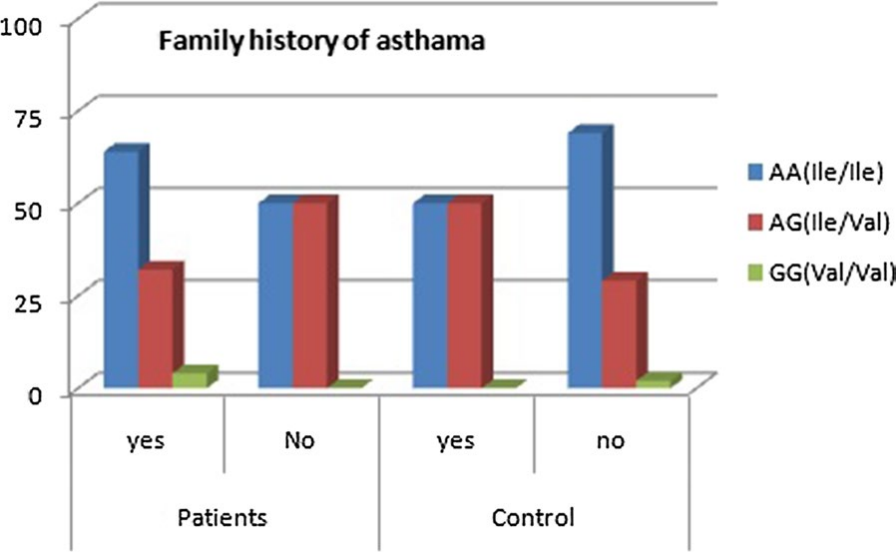
The family history with genotype frequencies

The PCR amplification showed a band of 176 bp in all the samples (Fig. 1). The resultant 176-bp fragment digested with BsamA1 which identify the A–G transition (Fig. 2), showed an undigested 176-bp fragment for homozygotes Ile105-encoding allele (GSTP1 Ile105/Ile105 genotype); two fragments of 91 and 85 bp was seen for homozygous Val105-encoding individuals (GSTP1Val105/Val105 genotype) resulted. The PCR products from heterozygotes (GSTP1 Ile105/Val105 genotype) comprised 176, 91, and 85 bp fragments (Fig. 3). Homozygous genotype (Ile/Ile) was detected in 62% of asthmatic patients and in 68% of the controls whereas the Val/Val genotype becomes determined with just 4% of patients and 2% of controls. Further, the heterozygous genotype Ile/Val was identified in 34% of sufferers and 30% of controls. Overall, there was no significant difference among all the groups (P ≤ 0.01) (Figs. 4, 5).

There had been no significance differences between cases with unique GSTP1 genotypes with regards to WBC count, eosinophilic count, HB count, and ESR ratio (Table 4). With regards to atopy, the study iterated the study stratification i.e., first, the people with IgE > 100 (40 cases) and people with IgE < 100 (60 cases) while for the second, those with a history of allergy (21 cases) (42%) and people with out such history (29 cases) (58%). Table 5 suggests GSTP1 genotype frequencies when it comes to IgE level. The frequencies for GSTP1 genotypes in subjects with IgE levels < 100 IU/ml and > 100 IU/ml had been no longer significantly different (P = 0.056). Figure 5 suggests the affiliation of GSTP1 genotypes with a family history of bronchial asthma. No distinct associations were found among the GSTP1 genotypes and atopic status.

**Table 4:** Laboratory blood cell counts of study samples

Laboratory results	GSTP1 genotype			Kruskal–Wallis test (P)
	Ile/Ile (65)	Ile/Val (32)	Val/Val (3)	
WBC				
Min–mix	4.50–18.40	4.50–18.40	8.70–18.40	1.464 (0.0586)
Mean ± SD	8.7538 ± 2.65389	9.6094 ± 3.55213	12.3667 ± 5.26530	
Hb				
Min–mix	9.40–15.20	10.40–15.20	11.50–15.20	0.843
Mean ± SD	12.0369 ± 1.4726	12.6219 ± 1.2868	13.9667 ± 2.13620	− 0.058
ESR				
Min–mix	5.00–63.00	5.00–63.00	13.00–63.00	0.100 (0.0.586)
Mean ± SD	22.6154 ± 14.10307	25.9688 ± 15.19228	31.0000 ± 27.78489	
Eosinophil				
Min–mix	2.00–9.00	2.00–9.00	4.00–6.00	0.629 (0.0.586)
Mean ± SD	4.9692 ± 2.08394	5.4688 ± 2.16995	4.6667 ± 1.15470

**Table 5:** IgE level according to genotype distributions for GSTP1 gene polymorphism in sample studies with asthma

IgE concentration with genotype frequencies				Significance
	Genotype frequencies			
	AA (Ile/Ile)	AG (Ile/Val)	GG (Val/Val)	
Less 100	30	9	1	0.056
More 100	39	19	2	

## Discussion

Asthma is an intricate, multifactorial disease with appearance from genetic predisposition, immunological responses, and intermediate of environmental elements. It recollects as a foremost public health challenge that affects 100–150 million human beings across the globe [16]. In the current study, the incidence of bronchial asthma is higher among females than males in alignment with the earlier research [17]. The reasons for gender difference is unknown but there has been a connection with immunological and hormonal factors, and/or to variations in gender-specific responses to vocational and environmental exposures [18]. Genetic polymorphisms are also encouraged through gender. Immunoglobulin E (IgE) titer and asthma had been related to Single Nucleotide Polymorphisms (SNPs) in Thymic Stromal Lympho poietin (TSLP) [19].

Many research articles demonstrated the association of genes with hypersensitive reaction/asthma [20]. Ober et al. [21] cited the association of genes with bronchial asthma or atopy in more than 10 research articles. Most genetic studies conducted on asthma concentrated on genes in the chromosomes 11q and 5q and their association with the key bronchial asthma-associated phenotypes of Bronchial Hyper Responsiveness (BHR) and atopy. Although bronchial asthma is characterized through airway infection, oxidative stress is a crucial issue in this regard since few research inferred that there are genes involved in opposition to this action. GST gene polymorphisms acknowledged risk factors for some environmentally-associated diseases. Several population studies connected genetic variation in human GSTP1 with greater susceptibility to asthma and the severity of signs. An association study was designed in the literature to understand whether an allelic variation on the glutathione-S-transferase GSTP1 locus, affects the expression of BHR and atopy phenotypes in bronchial asthma. The enzyme encoded by GSTP1 makes use of a ramification of lipid and DNA products of oxidative pressure whereas the polymorphic editions of this gene are related with the enzyme’s altered catalytic function [9, 16].

As it evident from the literature that a number of study on GSTP1 polymorphism and its relation with a different aspect of the respiratory function of human is done. Mutation on GSTP1 does not have only direct effect to asthma symptoms but two-way gene-air pollution interaction between GSTP1 has the risk of childhood asthma. The study suggests that interaction between GSTP1 and PM10 may alter the susceptibility to childhood asthma and buffers the harmful effects of air pollution [22]. A significant interaction of GSTP1 SNPs, to atopy, and ETS exposure have been identified in a cohort study [23]. Reports from Tunisian children demonstrated that polymorphisms of GST genes were associated with asthma and atopy and its genotypes were considered useful in future treatment optimization in the cases of increasing risk profile of asthma [24]. Allelic frequencies of SNPs of GSTP1 was found statistically significantly different in the asthmatic group compared with the controls and also a relationship between allele frequencies and different clinical phenotypes such as atopia nocturnal dyspnea, and steroid dependency was observed in asthmatic patients suggesting GSTP1 role in severeness of airway dysfunction [25]. As it is now well indicated in different studies that GSTP1 genotypes and polymorphism have important roles in asthma pathogenesis. It is also possible that intermediate metabolites, which are not metabolized or not excreted due to malfunction of GSTP1 may damage cells contributing to the pathogenesis of asthma [26].

The current study determined that there is a dramatic decrease in the frequency of GSTP1 Val105/Val105 in asthmatic and control subjects. The presence of genotypes GSTP1 Ile105/Ile105 and Il105/Val105 were found in 62% of the sufferers and 68% of the controls as well as in 34% of patients and 30% of controls respectively with no significant difference among the groups (P ≤ 0.01). According to Table 5, after correction for allele version with age, gender and different parameters that were consistent with laboratory outcomes of character traits, the frequency of GSTP1 Val105/Val105 correlated with increasing WBC (8.70–18.40) when compared with different allele variations that had less number of WBCs (Table 5). The increasing values of HB, ESR and IgE < 100 were correlating with decreasing values of eosinophil rapprochement with other allele contrast (Table 5).

So, the genotype distributions of GSTP1 gene polymorphism between the asthma subjects and controls showed no significant difference (P = 0.056). In most human beings, the major GSTP1 alleles are Ile105 (frequency > 50%). Epidemiological studies advised that due to individual differences within the expression of allelic variations of GSTP1 gene, they may be differences in slobber to numerous chemical substances and differences in capability to metabolize these retailers [27]. The higher IgE level, within the control group, can be explained through the better occurrence of parasitic infection and affected the patients from the region under study [28]. Some of the genes related to bronchial asthma have been replicated in one-of-its-kind research, but a maximum of them are not investigated further. This may be due to the fact that different investigations were conducted among different populations with exclusive genetic heritage, age, time, gender and environmental features.

## Conclusion

To the best of the researcher’s knowledge, this is the first-of-its-kind report with regards to the role played by GSTP1 polymorphisms in bronchial asthma among Iraqi patients. The findings of the current study no longer assist in the critical function of GSTP1 gene polymorphism within the evolution of asthma disorder. So a much wider and in-depth investigation of genetic–environmental interplay is required. Therefore, future studies ought to be executed in greater realistic settings with much environmental exposure so as to reach an authenticated starting point for the detection and discussion of gene–environment interplay and other genes concerned with the antioxidant pathway.
